# Integration of lipidomics and transcriptomics data towards a systems biology model of sphingolipid metabolism

**DOI:** 10.1186/1752-0509-5-26

**Published:** 2011-02-08

**Authors:** Shakti Gupta, Mano R Maurya, Alfred H Merrill Jr, Christopher K Glass, Shankar Subramaniam

**Affiliations:** 1 Department of Bioengineering, University of California, San Diego, 9500 Gilman Dr., La Jolla CA 92093, USA; 2 School of Biology & Petit Institute for Bioengineering and Bioscience, Georgia Institute of Technology, Atlanta, GA 30332-0230, USA; 3 Department of Cellular and Molecular Medicine, University of California, San Diego, 9500 Gilman Dr., La Jolla CA 92093, USA; 4 Department of Chemistry & Biochemistry, San Diego Supercomputer Center and Graduate Program in Bioinformatics, University of California, San Diego, 9500 Gilman Dr., La Jolla CA 92093, USA

## Abstract

**Background:**

Sphingolipids play important roles in cell structure and function as well as in the pathophysiology of many diseases. Many of the intermediates of sphingolipid biosynthesis are highly bioactive and sometimes have antagonistic activities, for example, ceramide promotes apoptosis whereas sphingosine-1-phosphate can inhibit apoptosis and induce cell growth; therefore, quantification of the metabolites and modeling of the sphingolipid network is imperative for an understanding of sphingolipid biology.

**Results:**

In this direction, the LIPID MAPS Consortium is developing methods to quantitate the sphingolipid metabolites in mammalian cells and is investigating their application to studies of the activation of the RAW264.7 macrophage cell by a chemically defined endotoxin, Kdo_2_-Lipid A. Herein, we describe a model for the C_16_-branch of sphingolipid metabolism (i.e., for ceramides with palmitate as the N-acyl-linked fatty acid, which is selected because it is a major subspecies for all categories of complex sphingolipids in RAW264.7 cells) integrating lipidomics and transcriptomics data and using a two-step matrix-based approach to estimate the rate constants from experimental data. The rate constants obtained from the first step are further refined using generalized constrained nonlinear optimization. The resulting model fits the experimental data for all species. The robustness of the model is validated through parametric sensitivity analysis.

**Conclusions:**

A quantitative model of the sphigolipid pathway is developed by integrating metabolomics and transcriptomics data with legacy knowledge. The model could be used to design experimental studies of how genetic and pharmacological perturbations alter the flux through this important lipid biosynthetic pathway.

## Background

Sphingolipids (SL) are categorized as lipids with a sphingoid base backbone [1] that is often derivatized with an amide-linked fatty acid to make ceramides (Cer) and more structurally complex SL with diverse biological functions [2]. SL in essentially every subcategory, from the lipid backbones [3] to complex SL [4], are highly bioactive and play important roles in diseases [5,6]; hence, methods for "lipidomic" analysis of SL and SL metabolism are important for an in-depth understanding of these enigmatic compounds. In recent years, a number of large-scale experimental and bioinformatics projects have begun to address the complexity of the lipidome. Examples include the Lipid Metabolites and Pathways Strategy (LIPID MAPS) Consortium [7], The Lipid Library [8], CYBERLIPID CENTER [9] and LipidBank [10]. In particular, LIPID MAPS has become a comprehensive resource for information on classification, structures and quantitative data on lipids and provides an opportunity for developing quantitative models of lipid synthesis and metabolism thus facilitating a mechanistic and systems-level understanding.

The de novo biosynthesis of SL begins with production of the sphingoid base, which utilizes serine and palmitoyl-coenzyme A (CoA) and various fatty acyl-CoAs to make N-acylsphinganines (dihydroceramides, DHCer) that are desaturated to Cer (N-acylsphingosines) and incorporated into more complex SL such as ceramide 1-phosphate (CerP), sphingomyelin (SM), glucosyl- and galactosyl-ceramide (GlcCer and GalCer) and more complex glycosphingolipids [2,11]. Ceramide can also be synthesized by recycling of sphingosine from turnover of SL such as SM [11,12]; furthermore, sphingosine and sphinganine are phosphorylated to sphingosine 1-phosphate (S1P) and sphinganine 1-phosphate (DHSph1P) which are intermediates of sphingoid base degradation [13] and cell signaling molecules [14].

Due to the complexity of this pathway, and the paucity of data for its many metabolites, there are only a few models of SL metabolism available in the literature [15-18]. The LIPID MAPS Consortium [7] has quantified the global changes in lipid metabolites and genes in RAW 264.7 macrophage cells treated with Kdo_2_-Lipid A (KLA). KLA is the active component of inflammatory lipopolysaccharide which functions as a selective agonist of Toll-like receptor 4 (TLR4) and mimics bacterial infection. The measurements are carried out over a 24-hour time period and the data is freely available via the LIPID MAPS website [7]. The goal of the work presented here is to develop a predictive kinetic model for SL metabolism using the lipidomics and transcriptomics data from the LIPID MAPS studies. This manuscript is organized as follows: we first briefly discuss the experimental data preprocessing and the methodology used for estimating the rate parameters, then we present the results of parameter estimation, followed by discussion and conclusions.

## Methods

### Network simplification

**Figure 1:**
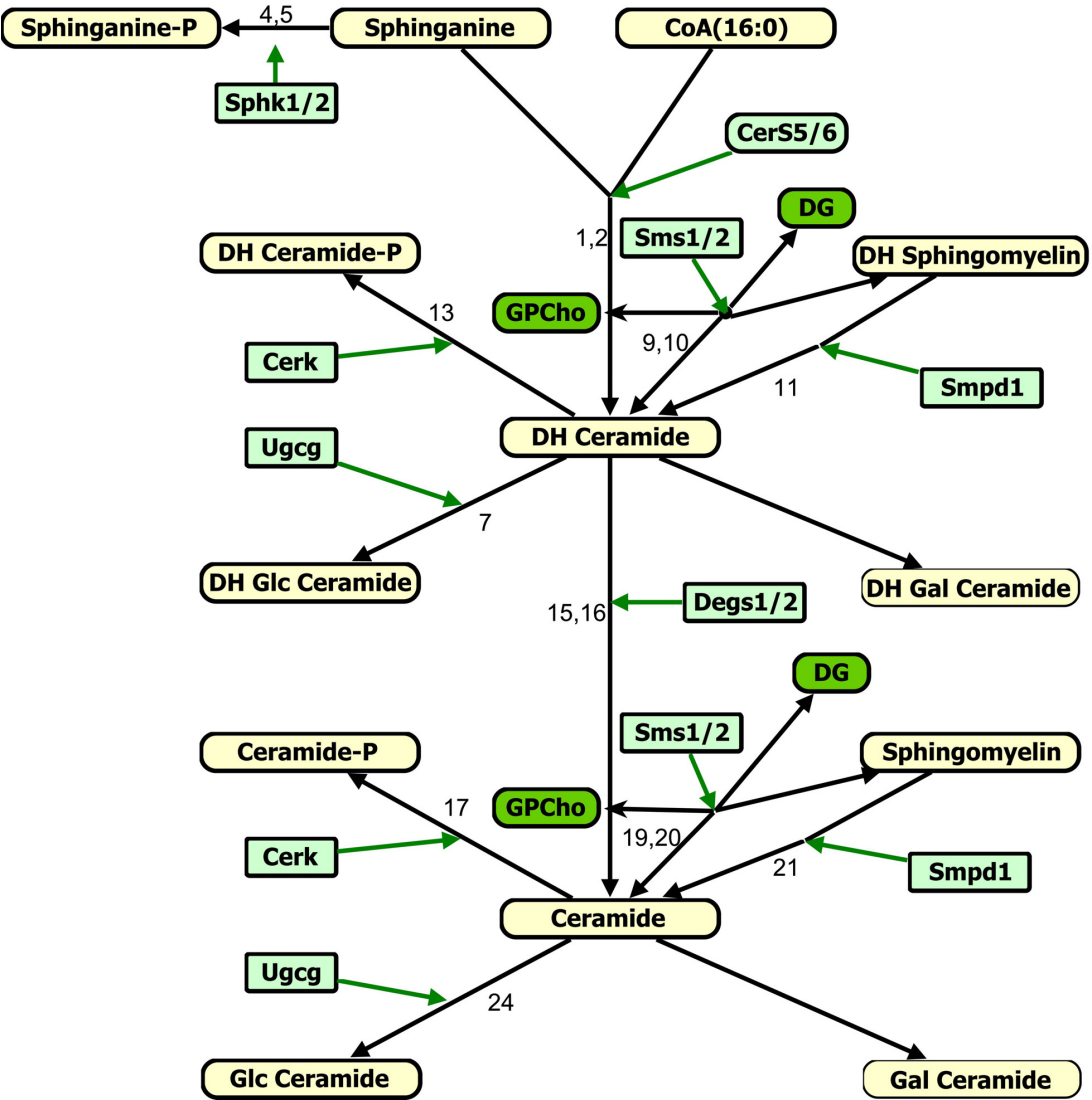
**Kdo_2_-Lipid A (KLA) stimulated sphingolipid metabolism pathway**. The numbers above (or near) the arrows are reaction numbers (Table 1). The default degradation reactions are not labeled. Metabolites and enzymes are represented as rectangular boxes with round corner and rectangular boxes, respectively. The measured and unmeasured metabolites that are and are not present in detectable amounts are differentiated by thick and thin borders, respectively. For full name of metabolites, see the list of abbreviations, presented at the end of the manuscript.

A detailed metabolic reaction network was developed using the information available in the literature and the KEGG pathways database [19] (Figure 1). The C_16_-branch of Cer biosynthesis (i.e., the Cer and DHCer with palmitate as the N-acyl-linked fatty acid) was selected for developing the model because this is a major subspecies for all categories of complex SL in the RAW264.7 cells. VANTED software was used to draw the reaction network [20]. It is common in modeling studies for the network to contain several unmeasured nodes (e.g. metabolites and genes); in our pathway (Figure 1), quantities are known for all of the metabolites and genes except DHGalCer and GalCer (which are present in such small amounts that they are below the limit of detection until the cells are activated) and are expressed as leaf-nodes (last metabolite in each branch) in the network. One of the steps in our matrix-based fast algorithm for parameter-estimation requires experimental data on all metabolites except on the leaf-nodes/metabolites in the network. A detailed procedure for simplifying the network, if the network contains unmeasured components, is described in our previous work [21]. The leaf-nodes were exempted from the model described in this paper because the reactions leading to the unmeasured leaf metabolites were combined with the default degradation of their precursors.

### Experimental data and preprocessing

The LIPID MAPS Consortium has measured all the major lipids in mouse macrophage RAW 264.7 cells grown in 10% serum and treated with KLA. All metabolites were measured in pmol/μg-DNA units. The time-courses of lipids and genes were measured under two conditions: (a) normal condition designated as control and (b) inflamed condition (stimulation by KLA). Time series comprising of 8 data points at 0, 0.5, 1, 2, 4, 8, 12 and 24 hr were measured with three biological replicates consisting of three technical replicates each. The three technical replicate experiments were performed on the same day with a single batch of cells. In addition, each time course was repeated three times on different days with different batches of cells (biological replicates). For kinetic modeling purposes, outlier points were detected by a t-test and were excluded at each time-point. The resulting data from all the replicates were averaged at each time-point. Data was processed for all metabolites under the treatment with KLA and control conditions.

Serine palmitoyltransferase activity *in vitro *was determined using the assay conditions described previously [22] but with [U-^13^C]palmitoyl-CoA as the labeled substrate. After extraction of the products, the amounts of [^13^C]3-ketosphinganine were determined by liquid chromatography, electrospray-ionization tandem mass spectrometry [23]. The assay was conducted using 100 μg of protein obtained by sonication of RAW264.7 cells in the buffer used for the assay.

### Development of a kinetic model and parameter estimation

We have developed a kinetic model of the SL metabolism. The procedure is similar to the one described in our previous work [21,24], but it is presented here through an example of reactions from the SL network for completeness. The reaction rates were described by linear or law of mass action kinetics with the assumption that for enzymatic reactions, the substrate concentrations are much smaller as compared to the corresponding Michaelis constant, K_m_. For example, the following types of reaction schemes and rate expressions were used:

$$\begin{array}{ll} \text{Reaction} & \text{Rate expressions}\\ \text{A}\to \text{B} & \text{k}*[\text{A}]\\ \text{A}+\text{B}\to \text{A}.\text{B} & \text{k}*[\text{A}]*[\text{B}]\\ {}[\text{Enz}]\text{A}\to \text{B} & \text{k}*[\text{Enz}]*[\text{A}] \end{array}$$

The enzymes involved in SL metabolism can be regulated at multiple levels from mRNA expression to posttranslational modification. mRNA data on the genes involved in the pathway is available from microarray experiments (Additional file 1 Table S1). However, the corresponding proteomics data is not available. Hence, in our modeling approach, we have captured the effect of temporal changes in transcription and protein levels by utilizing the microarray data with a three hour time delay as an input to the model. This assumes that the corresponding protein profile is similar to the mRNA time-course with the three hour delay, i.e., p(t) = g(t-3) where p(t) and g(t) denote the level of protein and gene-mRNA, respectively, at time t which is in hrs. A three hour time-delay was chosen based on the general consensus on delay in the protein synthesis from mRNA [25]. Here is an example of model formulation; the enzymes Sphk1 and Sphk2 catalyze the conversion of DHSph into DHSph1P (Figure 1). This effect was functionally captured through the reactions:

$$\begin{array}{cc} {}[\text{Sphk1}]\;DHSph\overset{k_{\text{f}4}}{\to }\text{DHSph1P} & \text{For}\;\text{the}\;\text{activity}\;\text{of}\;\text{Sphk}1 \end{array}$$

$$\begin{array}{cc} {}[\text{Sphk2}]\;DHSph\overset{k_{\text{f}5}}{\to }\text{DHSph1P} & \text{For}\;\text{the}\;\text{activity}\;\text{of}\;\text{Sphk}2 \end{array}$$

The effective rate of DHSph1P production was written as (k_f4_×[Sphk1] + k_f5_× [Sphk2])×[DHSph]. The flux expressions obtained from this scheme were linear in rate parameters and nonlinear in metabolite concentrations. The matrix-based approach to estimate the rate constants is described below in terms of the reaction numbers labeled in Figure 1 and listed in Table 1. Eq. 1 describes the rate of change of [DHSph1P] and [C_16 _DHGlcCer].

(1) d[DHSph1P]dt=kf4[DHSph][Sphk1]+kf5[DHSph][Sphk2]− kf6[DHSph1P]d[C16 DHGlcCer]dt=kf7[C16 DHCer][Ugcg]−kf8[C16 DHGlcCer]

**Table 1:** The estimated values of the rate parameters in the model of sphingolipid metabolism.

**No**.	Reactions	Parameter Names	Values
1	DHSph + CoA16 + CerS6 → C_16 _DHCer	k_f1_	1.30E+01 ± 1.01E-02
2	DHSph + CoA16 → C_16 _DHCer	k_f2_	5.62E-02 ± 1.30E-02
3	C_16 _DHCer →	k_f3_	1.11E-03 ± 3.20E-03
4	DHSph + Sphk1 → DHSph1P	k_f4_	1.49E-04 ± 1.11E-03
5	DHSph + Sphk2 → DHSph1P	k_f5_	1.61E-02 ± 8.38E-04
6	DHSph1P →	k_f6_	5.28E-01 ± 1.83E-02
7	C_16 _DHCer + Ugcg → C_16 _DHGlcCer	k_f7_	1.81E-02 ± 2.71E-03
8	C_16 _DHGlcCer →	k_f8_	2.99E-01 ± 3.25E-02
9	C_16 _DHCer + Sms1 + C_16 _GPCho ←→	k_f9_	1.15E-01 ± 6.85E-03
	C_16 _DHSM + Sms1 + C_16 _DG	k_b9_	1.7176 ± 5.47E-03
10	C_16 _DHCer + Sms2 + C_16 _GPCho ←→	k_f10_	3.88E-01 ± 1.02E-02
	C_16 _DHSM + Sms2 + C_16 _DG	k_b10_	8.63E-01 ± 1.23E-02
11	C_16 _DHSM + Smpd1 → C_16 _DHCer	k_f11_	1.07E-01 ± 5.62E-03
12	C_16 _DHSM →	k_f12_	2.49E-02 ± 3.01E-03
13	C_16 _DHCer + Cerk → C_16 _DHCerP	k_f13_	5.00E-05 ± 7.20E-06
14	C_16 _DHCerP →	k_f14_	8.25E-02 ± 6.57E-03
15	C_16 _DHCer + Degs1 → C_16 _Cer	k_f15_	9.07E-03 ± 9.56E-03
16	C_16 _DHCer → C_16 _Cer	k_f16_	8.03E-01 ± 1.39E-02
17	C_16 _Cer + Cerk → C_16 _CerP	k_f17_	1.29E-04 ± 7.64E-06
18	C_16 _CerP →	k_f18_	1.12E-01 ± 4.05E-03
19	C_16 _Cer + Sms1 + C_16 _GPCho ←→	k_f19_	6.50E-02 ± 3.13E-02
	C_16 _SM + Sms1 + C_16 _DG	k_b19_	4.72E-02 ± 8.67E-03
20	C_16 _Cer + Sms2 + C_16 _GPCho ←→	k_f20_	4.48E-01 ± 2.50E-02
	C_16 _SM + Sms2 + C_16 _DG	k_b20_	1.3343 ± 2.52E-02
21	C_16 _SM + Smpd1 → C_16 _Cer	k_f21_	1.91E-05 ± 1.98E-02
22	C_16 _SM →	k_f22_	9.79E-03 ± 1.28E-03
23	C_16 _Cer →	k_f23_	0.00E+00 ± 2.55E-03
24	C_16 _Cer + Ugcg → C_16 _GlcCer	k_f24_	2.39E-02 ± 3.05E-03
25	C_16 _GlcCer →	k_f25_	4.58E-03 ± 3.43E-03

Microarray data was used for protein concentration.

X → means default degradation of the metabolite X.

Unit of first order reaction is 1/hr.

Unit of second order reaction is 1/hr when it involves protein as a modifier as we have used fold change data with respect to control for these variables.

Unit of third order reaction is μg DNA/(pmol * hr) when it involves protein as a modifier.

Unit of third order reaction is μg DNA/(Ratio Int * hr) when it involves DG and protein as modifiers.

where the rate constants *k_i _*(i = f4, f5, f6, f7, f8) are as defined in Table 1.

If the metabolite concentrations were known and the rate parameters were unknown, then the ordinary differential equations (ODEs) can be rearranged in a matrix format as shown in Eq. 2.

(2) $$\begin{array}{l} \left[\begin{array}{l} \frac{\text{d[DHSph1P]}}{\text{dt}}\\ \frac{{\text{d[C}}_{\text{16}}\text{DHGlcCer]}}{\text{dt}} \end{array}\right]={\left[\begin{array}{ll} \text{[DHSph][Sphk1]} & 0\\ \text{[DHSph][Sphk2]} & 0\\ -\text{[DHSph1P]} & 0\\ 0 & {\text{[C}}_{\text{16}}\text{DHCer]}\;\text{[Ugcg]}\\ 0 & -{\text{[C}}_{\text{16}}\text{DHGlcCer]} \end{array}\right]}^{T}\left[\begin{array}{l} {\text{k}}_{\text{f4}}\\ {\text{k}}_{\text{f5}}\\ {\text{k}}_{\text{f}6}\\ {\text{k}}_{\text{f}7}\\ {\text{k}}_{\text{f}8} \end{array}\right]\\ \text{Y}=\;\;\text{X}\;*\;\;\;\text{b} \end{array}$$

The coefficients in the matrix **X **are linear/nonlinear functions of metabolite and gene concentrations. All the equations used in the simulation are listed in Appendix A. **X **is completely defined. The left hand side of the equations (matrix **Y**) was computed using discretization and the experimental data (Eq. 3).

(3) $${\frac{dx}{dt}|}_{t=t_{k}}=\frac{{x|}_{t=t_{k}}-{x|}_{t=t_{k-1}}}{t_{k}-t_{k-1}}$$

Eq. 2 contains known matrices **X **and **Y**, and the only unknown in this equation is the rate-constant vector **b**. The constrained least-squares approach (Matlab^® ^[26] function *lsqlin*) was used to solve **b**. *lsqlin *optimized the solution with objective function (Eq. 4) with additional constraints that all parameter values have to be positive.

(4) $$\min{\left\|\text{Y}-\text{Xb}\right\|}^{2}\;\text{where}\;\text{Y}\;\text{and}\;\text{Xb}\;\text{are:}{\frac{dy}{dt}|}_{\exp}\text{and }{\frac{dy}{dt}|}_{pred}$$

The estimated values of the parameters were further refined by using generalized constrained nonlinear optimization (Matlab^® ^function *fmincon*) where the objective (Eq. 5) was to minimize the weighted fit-error between the experimental and predicted metabolite concentrations. The algorithm of *fmincon *does not require a matrix form. Thus, numerical integration was used (e.g. Matlab^® ^function *ode23*) to simulate the system to circumvent the discretization error. The combined use of *lsqlin *and *fmincon *made the overall process computationally efficient. The objective function for use with *fmincon *was:

(5) $$\begin{array}{l} \underset{K,X_{0}}{\min}\sum_{i=1}^{nsp}\left(\sum_{j=1}^{nt}{\left(y_{i,j,\exp}-y_{i,j,pred}(K,X_{0})\right)}^{2}\right)\\ K:\text{ prameters (rate constants)}\\ X_{0}\text{: Initial conditions (species concentrations)} \end{array}$$

where, *nt *is the number of time-points and *nsp *is the number of species.

The initial concentrations of the metabolites were also optimized in a narrow range around the experimental values. When data on more than one condition was available, then all the data was used to compute the fit-error by simulating the model several times individually and minimizing the objective function collectively.

### Estimation of uncertainty in the optimized parameters

The variation among the different technical and biological replicates of lipid and gene data should be accounted for in the estimated values of the parameters. Hence, an uncertainty analysis was performed on the parameters. Their standard error of mean (SEM) was computed as follows:

1. Compute the SEM in the lipid and gene data at each time point (Additional file 1 Table S1b).

2. Create a candidate data set for parameter estimation by generating *nsp *x *m *random matrix utilizing the normal distribution, scale it with the corresponding SEM and add the scaled-value matrix to the mean-value data on lipids and genes (Additional file 1 Table S1a).

3. Estimate the parameters using the candidate data set thus producing a one parameter-value set.

4. Repeat steps 2-3 k times to generate k parameter-value sets (k = 10 in our simulation).

5. Compute the SEM for each parameter across the k sets.

## Results

### Fit to experimental data

**Figure 2:**
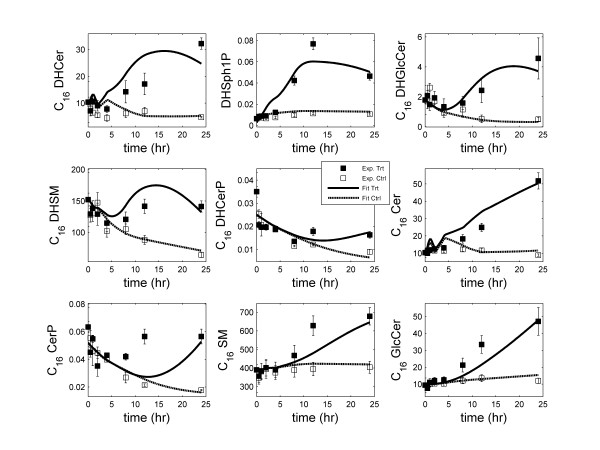
**Simulation results of kinetic modeling of sphingolipids network: Fit of the predicted response (control and treatment with KLA) to the corresponding experimental data**. In the legend, 'Ctrl' refers to control and 'Trt' refers to KLA treatment of RAW 264.7 cells. The error-bars shown on the experimental data are standard-error of mean.

ODEs were generated for all metabolites in the network and effective rate constants were estimated for the simplified SL reaction network using the approach described in the "Methods" section. Table 1 lists the reactions and the corresponding estimated reaction-rate parameters included in the model. Lipid metabolism and signaling are complex processes and the mechanisms involved are only partially known. KLA treatment generated increases in most sphingolipids in RAW cells. The increase in sphinganine (DHSph), which doubled in 4 hours, followed by increases in downstream metabolites, including *N*-acyl-sphinganines (dihydroceramides, DHCer) after a lag of approximately 2 to 4 hours indicted the induction of *de novo *sphingolipid biosynthesis. To account the effect of KLA signaling, the time-courses of the concentrations of DHSph, CoA16, C_16 _DG, C_16 _GPCho and microarray data were used as input to the network (Additional file 1 Figure S1, Table S1). Linear interpolations of these time-courses were used in the integration. The decline in SL control experiment data suggested that the control experiments were not at steady state [7]. The assumption of steady-state was circumvented by including (fitting) data obtained in two experimental scenarios, namely, the treatment with KLA and the corresponding control data set, during parameter-estimation. The microarray data was used to represent the corresponding protein concentration with 3 hr time delay. For microarray data, fold change with respect to the control was used in the simulation corresponding to the treatment experiment (Additional file 1 Table S1). A fold-value of 1 was used in the simulation corresponding to the control experiment. CerS5 and Degs2 did not show significant change with respect to the control in microarray data, thus the corresponding reactions (reaction 2 and 14) were written without these enzymes. The omission of these enzymes indicated their constant activity in these reactions. The simplified model is a reliable predictive model as evidenced by the good fit to experimental data for most metabolites (Figure 2). For C_16 _DHCer (top-left panel), the fit is good up to 8 hrs after which it deteriorates. One possible reason for this deterioration in the fit is the discrepancy between mRNA and protein levels for the enzymes CerS5/6.

There were two problems in the optimization of parameters: (1) the wide range of metabolite concentrations, and (2) irregular time intervals (longer intervals at later times). The concentration of metabolites varied between 0.01 pmol/μg DNA (for C_16 _DHCerP) and 700 pmol/μg DNA (for C_16 _SM). Due to the orders of magnitude difference in the metabolite concentrations, the fits were biased towards metabolites with high concentration and resulted in a poor fit for the metabolites with lower concentrations. To resolve this issue, the experimental values and predicted values for each metabolite were scaled/normalized by its maximum experimental value. Then, the sum of squares of the normalized fit-error on all metabolites was minimized. This scaling, essentially, normalized the maximum experimental concentrations to 1 for all metabolites and resulted in approximately equal weight for all metabolites. Further, the data measured at irregular time intervals (12 hr difference in last two measurements) also created a problem in optimization and led to relatively poor fit at later time points. From parameter-estimation view-point, the measurements should be made at equal intervals so that equal weight is assigned to the entire time-course. To account for this, the point-wise-error was scaled by the 1/4 power of the length of the corresponding time-interval, resulting in a higher weight for later time points. Consequently, the quality of fit for the later time-points was improved. For most time points, the difference between the predicted and experimental data was within the standard-error of mean (Figure 2). The good fit was obtained for all metabolites under both treatment and control conditions.

### Parametric sensitivity and time-scale analysis

**Figure 3:**
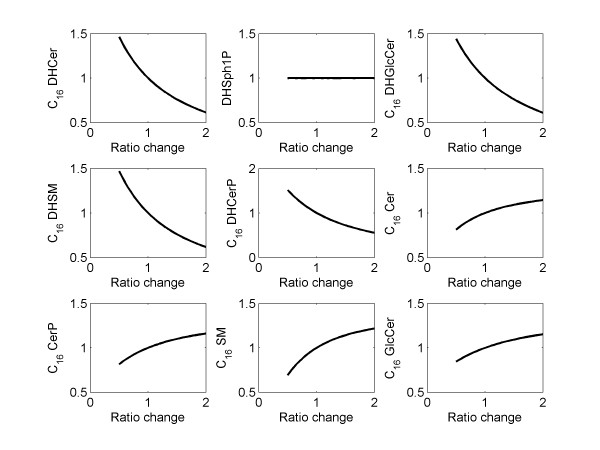
**Simulation results of parametric sensitivity analysis for the parameter k_f16 _(C_16 _DHCer → C_16 _Cer)**. X-axis: ratio of perturbed value of the parameter to the original (optimized) value of the parameter; Y-axis: fold-change in the maximum-value of state variables (metabolites).

*Parametric sensitivity analysis: *Parametric sensitivity analysis was performed by varying all parameters (one at a time) by two-fold up and down from its original (optimized) value. The sensitivity of each metabolite was studied by plotting *the fold-change at its maximum concentration as compared to the maximum concentration corresponding to the original value of the parameter *vs. *the ratio-change in the value of the perturbed parameter *(Figure 3). The numerical values of the sensitivity, i.e. the slope of the plots at the optimized value of the parameter, are listed in Additional file 1 Table S2. For each parameter and each metabolite, monotonic increase, decrease or no change was observed depending upon the respective location of the parameter and the metabolite chosen in the network. For example, an increase in the parameter k_f16 _(C_16 _DHCer → C_16 _Cer) (Figure 3) produced a decrease in all upstream metabolites except DHSph1P (Sphinganine-1P). An increase is observed for Cer (Figure 3 sensitivity ~ 0.25) and its downstream products such as Cer-1P, GlcCer, GalCer and SM. This is meaningful mechanistically because the increase in k_f16 _increases the flux of the reaction C_16 _DHCer → C_16 _Cer. If the level of C_16 _DHCer were not to change much, then one would expect almost proportional increase in C_16 _Cer (sensitivity ~ 1). However, this is not true because the level of C_16 _DHCer is reduced (sensitivity ~ -0.64). This results in a net sensitivity value of less than 1. Similarly, an increase in k_f16 _results in decreased DHCer-1P, DHGlcCer, and DHSM levels. These metabolites are products of DHCer and hence the sensitivities are consistent with the structure of the biochemical reaction network (Figure 1). Small to moderate sensitivities (Additional file 1 Table S2) suggest that the biochemical system is robust with respect to parametric perturbations.

*Uncertainty analysis on the parameters: *The SEM of the parameters is calculated as described in the Methods section and the results are reported in Table 1. Overall, the parameters are well-behaved and the parameter-estimation procedure is reliable because the fractional SEM for the lipid and gene data and the fractional SEM for most of the parameters are of the same order (20-30%).

*Time-scale analysis: *Time-scale is an important intrinsic property of dynamical biological systems. While the time-scale of metabolites, at which they evolve, can be gleaned by analyzing several time-courses under different conditions, a more systemic picture can be obtained through eigenvalue and eigenvector analysis of the Jacobian matrix of ordinary differential equations at the steady-state conditions. In our computations, the steady-state was identified by simulating the system corresponding to the control condition (no stimulus) for a long time (t = 1000 hr). The Jacobian matrix was computed through numerical differentiation of the expressions on the right hand sides of the ODEs with respect to the state variables. The eigenvalues were split into three broad ranges. For each eigenvalue, the metabolites with substantial contribution to the corresponding eigenvector were identified. Depending upon the eigenvalues and metabolites significantly contributing to the corresponding eigenvectors, these metabolites have been divided into three categories as listed in Table 2. When a metabolite contributed significantly in two eigenvectors spanning in two different eigen value ranges, the metabolite was assigned to the smaller eigen value because the fast manifold only determines its initial transients and the slow manifold governs the later response leading to steady state. A comparison of Table 2 and Figure 2 shows that the time scale of the metabolite is dependent on its location (whether it is leaf node metabolite or intermediate metabolite) and its concentration. The medium time-scale metabolites (column 2 in Table 2) are the leaf node metabolites having low concentration (~ 0.01-1 pmole/μg of DNA); the slow time-scale metabolites (column 3 in Table 2) are the leaf node metabolite with high concentration (~ 10^2^-10^3 ^pmole/μg of DNA). DHCer and Cer have fast time-scale because of their intermediate location in the network, moderate concentration (~10 pmole/μg of DNA) and high flux through these nodes in the production of SM.

**Table 2:** Results of eigenvalue based time-scale analysis of the metabolites.

Fast (~1 hr)	Medium (~10 hr)	Slow (~100 hr)
C_16 _DHCer	DHSph1P	C_16 _SM
C_16 _Cer	C_16 _DHGlcCer	C_16 _GlcCer
	C_16 _DHSM	
	C_16 _DHCerP	
	C_16 _CerP	

## Discussion

This study has used the large data sets from mass spectrometric measurements of the SL and the microarray data of the mRNAs of RAW264.7 cells generated by the LIPID MAPS Consortium to evaluate a model for SL metabolism.

### Importance of including the transcriptomics data and the data on fatty acyl-CoA

In this study, we have included the microarray data for the lipid-related genes using a time-delay of three-hours to account for the time for mRNA translation and protein translocation. The use of mRNA data for kinetic modeling was motivated by good correlations between specific genes and its metabolic products in the sphingolipid pathway [27]. In general, protein levels follow the qualitative profile of mRNA with appropriate time-delays. However, we note the potential caveat that the protein levels for some proteins may not be even qualitatively similar to the transcriptional levels of their genes. The discrepancy of mismatch between the mRNA and protein profiles can arise due to several factors such as dependence of mRNA translation on ribosome binding site (RBS) sequences, post translational modification of the protein, protein translocation and its stability. This may introduce errors in the estimated values of the kinetic parameters.

To delineate the importance of the transcriptional data, we first developed the mathematical model without using the gene/protein data. The rate parameters were estimated using the lipidomics data alone. We visualized the fit to experimental data (equivalent of the plots shown in Figure 2; data not shown). A reasonable fit was obtained for all metabolites except C_16_-DHCer. To resolve disagreement in the shape of DHCer, we identified the reactions in which these lipids were consumed or produced. We also analyzed the time-course of the mRNA levels for the genes related to these reactions. Most of the genes exhibited differential regulation at later time points (i.e., the ratio of data with KLA treatment to control being significantly different from 1). Among several differentially regulated genes, the prominent were CerS6 (reaction 1), Smpd1 (reactions 9 and 17), Degs1 (reaction 13) and Ugcg (reaction 20). The up-regulation and involvement of CerS6 in the production of DHCer suggested that the gene data must be included in the network to capture the DHCer dynamics. After adding the gene data with delay, we observed substantial improvement in the fit to the experimental data for DHCer (Figure 2).

The profile of palmitoyl-CoA16 also increases monotonically during 0-24 hrs (Additional file 1 Figure S1). Even though [DHSph] increases till 4 hr and comes back to control level at 24 hr, the influx to DHCer is maintained throughout 0-24 hr because of increase in palmitoyl-CoA and CerS6 at later time points. Due to this reason, DHCer shows a monotonically increasing profile during 0-24 hr. Thus, the profiles of CerS6 and palmitoyl-CoA are important to obtain a good fit on DHCer.

### Rate parameters for the enzymes

We compared the combined rate constant for the enzymes CerS5/CerS6 (pmol/hr/μg-DNA) with an estimation of the maximum flux through the de novo biosynthetic pathway based on the activity of serine palmitoyltransferase assayed in vitro with optimal concentrations of substrates, which was 30 + 1 pmole/min/mg protein for control (unstimulated) RAW264.7 cells. To convert the estimated values to the same units, we used estimations (by measurement) for the relationships between DNA, cell number and protein amount of ~3 μg DNA/10^6 ^RAW264.7 cells, and 10^6 ^cells have ~0.25 mg protein. Thus, the computed value for C16-SL biosynthesis by CerS5/CerS6 is ~1 pmol/min/mg protein, which is about one order of magnitude lower than the theoretical maximum rate of sphinganine production by the cells. This might mean that the calculated value is low due to inaccuracies in some of the modeling approximations, including the use of linear kinetics instead of Michaelis-Menten kinetics; however, the differences might be real because serine palmitoyltransferase has usually not been found to be operating at V_max _because its substrates are not saturating [28,29] and other CerS also contribute to the utilization of the sphinganine that is produced de novo. In addition, sphinganine and sphinganine 1-phosphate are elevated to some extent in RAW264.7 cells, which implies that CerSs are not trapping all of the sphinganine that is made.

### Similarity in the rate parameters for same gene/enzyme involved in different reactions

**Figure 4:**
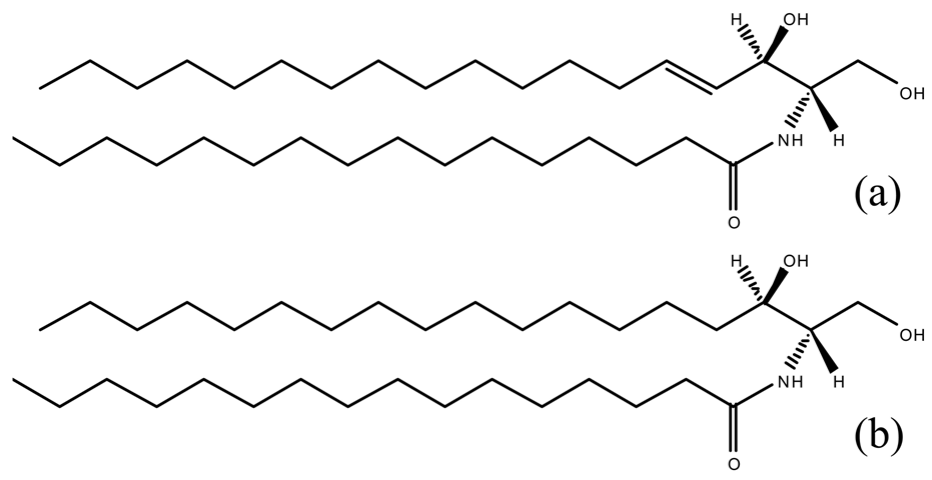
**Chemical structure of (a) C_16 _Cer and (b) C_16 _DHCer**.

**Figure 5:**
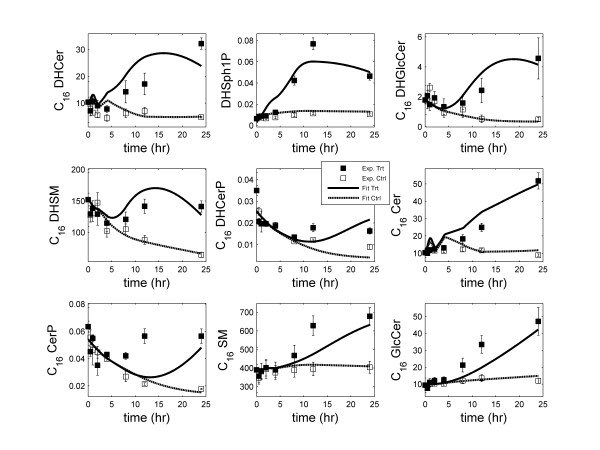
**Simulation results of kinetic modeling of sphingolipids network where parameters for CerK, Ugcg and SMS1/2 are same for the reactions involving Cer and DHCer: Fit of the predicted response (control and treatment with KLA) to the corresponding experimental data**. In the legend, 'Ctrl' refers to control and 'Trt' refers to KLA treatment of RAW 264.7 cells. The error-bars shown on the experimental data are standard-error of mean.

Structurally, DHCer and Cer differ by one double bond (Figure 4). However, both are converted to their corresponding derivatives by four genes, namely Ugcg, Cerk and Sms1/2 (Figure 1). To check the effects of this double bond on the rate parameter, we compared the rate parameters for the above genes/enzymes in their reactions. The rate parameter for SMS2 (k_f10 _and k_f20_) and Ugcg (k_f7 _and k_f24_) was found approximately similar for the both reactions involving DHCer and Cer. For Sms1 and Cerk, the rate parameters for the two reactions differed by a factor of 2 (Table 1). To further test whether we can find the common rate constants for these enzymes in their reactions, we carried out the optimization using same rate constant for these enzymes in their reactions. We were able to get reasonably good fit for all of the metabolites (Figure 5). In the modified optimization, the values obtained for these rate-parameters were between the corresponding values for the two reactions obtained in the original optimization (Additional file 1 Table S3). This result suggests that the affinities of these enzymes are similar for both the substrates Cer and DHCer.

### Consideration of different chain-lengths

C_16 _sphingolipids are used in this model because (1) these are present in higher amounts than the SL of larger chain-lengths, and (2) they showed significant differences between the treatment and control experiments in response to KLA. We can extend our model to include the metabolites with higher chain-lengths (e.g. C_18_, C_20_, C_22 _and C_24_). The reactions in the existing model (C_16_-based) will serve as a template that can be instantiated for higher chain-lengths. To estimate the rate parameters for the entire model, the parameters for the C_16_-based model can provide bounds for the rate parameters in the overall model for the similar types of reactions. For example, the rate constants for the reactions C_n _DHCer → C_n _DHGlcCer (where n = 18, 20, 22 and 24), can be constrained with a factor of 0.5 to 2 of the rate constant for the reaction C_16 _DHCer → C_16 _DHGlcCer in the model developed here.

### Use of the model for in-silico perturbation experiments

The model can be used to perform knockdown (KD) or other perturbation experiments in-silico. Such computational studies provide useful insights into the behavior of the system which can add in designing the actual perturbation experiments. These simulations can assist in finding the propagation of the effects of KD in the network and lead to a better design of experiments (e.g. when the measurements should be made). Simulation can also suggest whether a high level of KD is needed such that the differences between the KD and control scenarios would be statistically significant. The data generated from new perturbation experiments can be used to further refine the model. For the enzymes, the KD perturbations can be simulated by decreasing the corresponding rate parameter because amount of the (active) enzyme directly affects the rate parameters. The KD perturbation simulation results will be similar to the sensitivity analysis results on the corresponding rate parameter. For example, the effect of 50% KD of the enzyme for the reaction 16 (Figure 1 Degs2), can be predicted from the simulation for sensitivity analysis in which the value of the parameter *k*_f16 _is reduced by 50% (Figure 3).

The case of knockdown of Cerk (cermide kinase) is also interesting due to its direct effect on Ceramide-1-phosphate (CerP) which inhibits apoptosis and induces cell growth [30]. The result of sensitivity analysis for the corresponding rate parameter, k_f17 _(C_16 _Cer + Cerk → C_16 _CerP), is shown in Additional file 1 Figure S2. Decrease in k_f17 _results in a corresponding decrease in the CerP level. These changes have also been observed in recent experiments validating our parametric sensitivity analysis and the predictive ability of the model [31]. CerP has also been implicated in regulating the homeostasis of calcium [32] thereby affecting the activity of several signaling pathways. CerP and Cerk mediate the effect of cytokines to activate cytosolic phospholipase A2 (cPLA_2_) and cyclooxygenase 2 (COX-2), resulting in increased production of prostaglandin E2 (PGE2), a mediator of inflammation [33]. Hence, it has been hypothesized that Cerk could be a potential target for anti-inflammatory drugs [34,35].

## Conclusion

Use of systems biology approaches is becoming more common in the study of lipids to elucidate their functions and roles in human health and diseases such as arthritis and cancer. Systems biology has already been recognized as an indispensable tool in pathway-based drug discovery. Here we have applied a matrix-based approach to develop a dynamic model of SL metabolism by integrating legacy information on the lipid pathways with novel experimental data. The metabolic pathway was reconstructed using information from the KEGG database and the existing literature. Based upon the network map reconstructed, we have developed an ordinary differential equations-based mathematical model. Parameter-estimation used a two-step approach. In the first step, a matrix-based approach provided an initial guess. The parameter-values were further refined in the second step. The resulting model fitted the experimental data well for all species and demonstrated that the integrated metabolic and signaling network and the experimental data are consistent with each other. The robustness of the model parameters was also validated through sensitivity analysis. Though we have used this two-step approach previously and applied it to eicosanoid lipid pathway, the major distinction lies in its application to the SL metabolic pathway and the integration of transcriptomic data with the metabolomic data along with legacy knowledge to develop the kinetic model. Previous computational models of sphingolipid metabolism were for non-mammalian systems in which only a few metabolites were measured as compared to the total number of metabolites in the reaction network. In comparison, our model is based on a large amount of experimental time-course data where the concentrations of most metabolites and mRNA levels of genes in the network are measured. This provides a more context-specific model for RAW cells in particular and for mammalian cell systems in general.

## Abbreviations used

Cer: Ceramide; CerP: Ceramide-1-phosphate; Cerk: ceramide kinase; Degs1: degenerative spermatocyte homolog 1 (Drosophila); Degs2: degenerative spermatocyte homolog 2 (Drosophila), lipid desaturase; CerS5: ceramide synthase 5; CerS6: ceramide synthase 6; Smpd1: sphingomyelin phosphodiesterase 1, acid lysosomal; Sphk1: sphingosine kinase 1; Sphk2: sphingosine kinase 2; Sms1: sphingomyelin synthase 1; Sms2: sphingomyelin synthase 2; Ugcg: UDP-glucose ceramide glucosyltransferase; C16 Cer: N-(hexadecanoyl)- sphing-4-enine (C16 Ceramide); C16 CerP: N-(hexadecanoyl)- sphing-4-enine-1-phosphate (C16 Ceramide-1-phosphate); C16 GlcCer: N-(hexadecanoyl)-1-β- sphing-4-enine (C16 Glucosylceramide); C16 SM: N-(hexadecanoyl)- sphing-4-enine -1-phosphocholine (C16 Sphingomyelin); C16 DHCer: N-(hexadecanoyl)-sphinganine (C16 Dihydroceramide); C16 DHCerP: N-(hexadecanoyl)-sphinganine-1-phosphate; C16 DHGlcCer: N-(hexadecanoyl)-1-β-glucosyl-sphinganine; C16 DHSM: N-(hexadecanoyl)-sphinganine-1-phosphocholine; DHSph: Sphinganine; DHSph1P: Sphinganine-1-phosphate; DHGalCer: Dihydro Galactosylceramide; GalCer: Galactosylceramide;

## Authors' contributions

SG designed the simulations, wrote the computer program, analyzed the experimental data and the simulation results and wrote the manuscript. AHM assisted in refining the reaction network. MRM assisted in designing some of the simulations, wrote part of the computer program and contributed in writing the manuscript. AHM, CKG and SS assisted in revising the manuscript. Sphingolipid and enzyme activity measurements were carried out in the laboratory of AHM. Transcriptomics experiments were carried out in the laboratory of CKG. SS supervised the modeling study. All authors have read and approved the final manuscript.

## Appendix A

The flux expressions for the reactions shown in Figure 1 are as follows:

v_1 _= k_f1_[DHSph][CoA16][CerS6]

v_2 _= k_f2_[DHSph][CoA16]

v_3 _= k_f3_[C_16 _DHCer]

v_4 _= k_f4_[DHSph][Sphk1]

v_5 _= k_f5_[DHSph][Sphk2]

v_6 _= k_f6_[DHSph1P]

v_7 _= k_f7_[C_16 _DHCer][Ugcg]

v_8 _= k_f8_[C_16 _DHGlcCer]

v_9 _= k_f9_[C_16 _DHCer][Sms1][C_16 _GPCho] - k_b9_[C_16 _DHSM][Sms1][C_16 _DG]

v_10 _= k_f10_[C_16 _DHCer][Sms2][C_16 _GPCho] - k_b10_[C_16 _DHSM][Sms2][C_16 _DG]

v_11 _= k_f11_[C_16 _DHSM][Smpd1]

v_12 _= k_f12_[C_16 _DHSM]

v_13 _= k_f13_[C_16 _DHCer][Cerk]

v_14 _= k_f14_[C_16 _DHCerP]

v_15 _= k_f15_[C_16 _DHCer][Degs1]

v_16 _= k_f16_[C_16 _DHCer]

v_17 _= k_f17_[C_16 _Cer][Cerk]

v_18 _= k_f18_[C_16 _CerP]

v_19 _= k_f19_[C_16 _Cer][Sms1][C_16 _GPCho] - k_b19_[C_16 _SM][Sms1][C_16 _DG]

v_20 _= k_f20_[C_16 _Cer][Sms2][C_16 _GPCho] - k_b20_[C_16 _SM][Sms2][C_16 _DG]

v_21 _= k_f21_[C_16 _SM][Smpd1]

v_22 _= k_f22_[C_16 _SM]

v_23 _= k_f23_[C_16 _Cer]

v_24 _= k_f24_[C_16 _Cer][Ugcg]

v_25 _= k_f25_[C_16 _GlcCer]

The differential equations describing the rate of change of metabolite concentrations are:

d[C_16 _DHCer]/dt = v_1 _+ v_2 _- v_3 _- v_7 _- v_9 _- v_10 _+ v_11 _- v_13 _- v_15 _- v_16_

d[DHSph1P]/dt = v_4 _+ v_5 _- v_6_

d[C_16 _DHGlcCer]/dt = v_7 _- v_8_

d[C_16 _DHSM]/dt = v_9 _+ v_10 _- v_11 _- v_12_

d[C_16 _DHCerP]/dt = v_13 _- v_14_

d[C_16 _Cer]/dt = v_15 _+ v_16 _- v_17 _- v_19 _- v_20 _+ v_21 _- v_23 _- v_24_

d[C_16 _CerP]/dt = v_17 _- v_18_

d[C_16 _SM]/dt = v_19 _+ v_20 _- v_21 _- v_22_

d[C_16 _GlcCer]/dt = v_24 _- v_25_

## Supplementary Material

Additional file 1 **Supporting Material**. This file is in PDF format and contains additional Figures S1-S2 and additional Tables S1-S3. Click here for file
