# Supplementary material for: Integration of lipidomics and transcriptomics data towards a systems biology model of sphingolipid metabolism
Source: BMC Syst Biol. 2011 Feb 8;5:26. doi: 10.1186/1752-0509-5-26 (PMC3047436; doi:10.1186/1752-0509-5-26)
Supplement: Additional file 1 — Supporting Material. This file is in PDF format and contains additional Figures S1-S2 and additional Tables S1-S3. [file 1752-0509-5-26-S1.DOC]

Supporting material

To

Integration of lipidomics and transcriptomics data towards a systems biology model of sphingolipid metabolism

*Running title*: **Modeling of Sphingolipid pathway**

Shakti Gupta^1^, Mano R Maurya^1^, Alfred H Merrill, Jr^2^, Christopher K Glass^3^ and Shankar Subramaniam^*1, 3, 4^

^1^Department of Bioengineering

University of California, San Diego, 9500 Gilman Dr., La Jolla CA 92093, USA

^2^School of Biology & Petit Institute for Bioengineering and Bioscience

Georgia Institute of Technology, Atlanta, GA 30332-0230, USA

^3^Department of Cellular and Molecular Medicine

University of California, San Diego, 9500 Gilman Dr., La Jolla CA 92093, USA

^4^Department of Chemistry & Biochemistry, San Diego Supercomputer Center and Graduate Program in Bioinformatics

University of California, San Diego, 9500 Gilman Dr., La Jolla CA 92093, USA

E-mail: [shakti@sdsc.edu](mailto:shakti@sdsc.edu), [mano@sdsc.edu](mailto:mano@sdsc.edu), [al.merrill@biology.gatech.edu](mailto:al.merrill@biology.gatech.edu), [ckg@ucsd.edu](mailto:ckg@ucsd.edu), [shankar@ucsd.edu](mailto:shankar@ucsd.edu)

^*^Corresponding Author: [Shankar@ucsd.edu](mailto:Shankar@ucsd.edu)


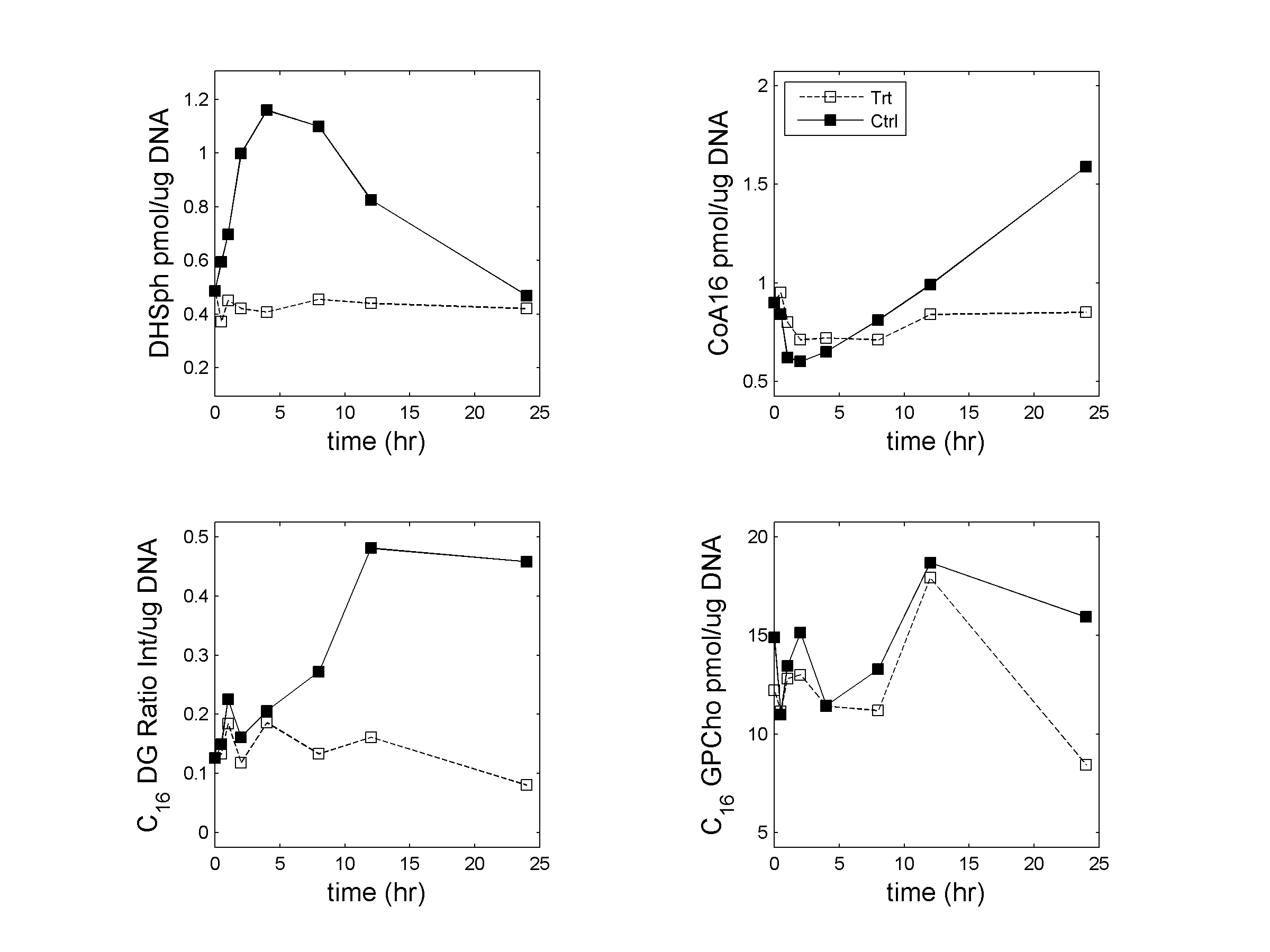


**Figure S1.** Experimental data for metabolites used in the simulation as input in the network. In the legend, ‘Ctrl’ refers to control and ‘Trt’ refers to KLA treatment of RAW 264.7 cells.

**
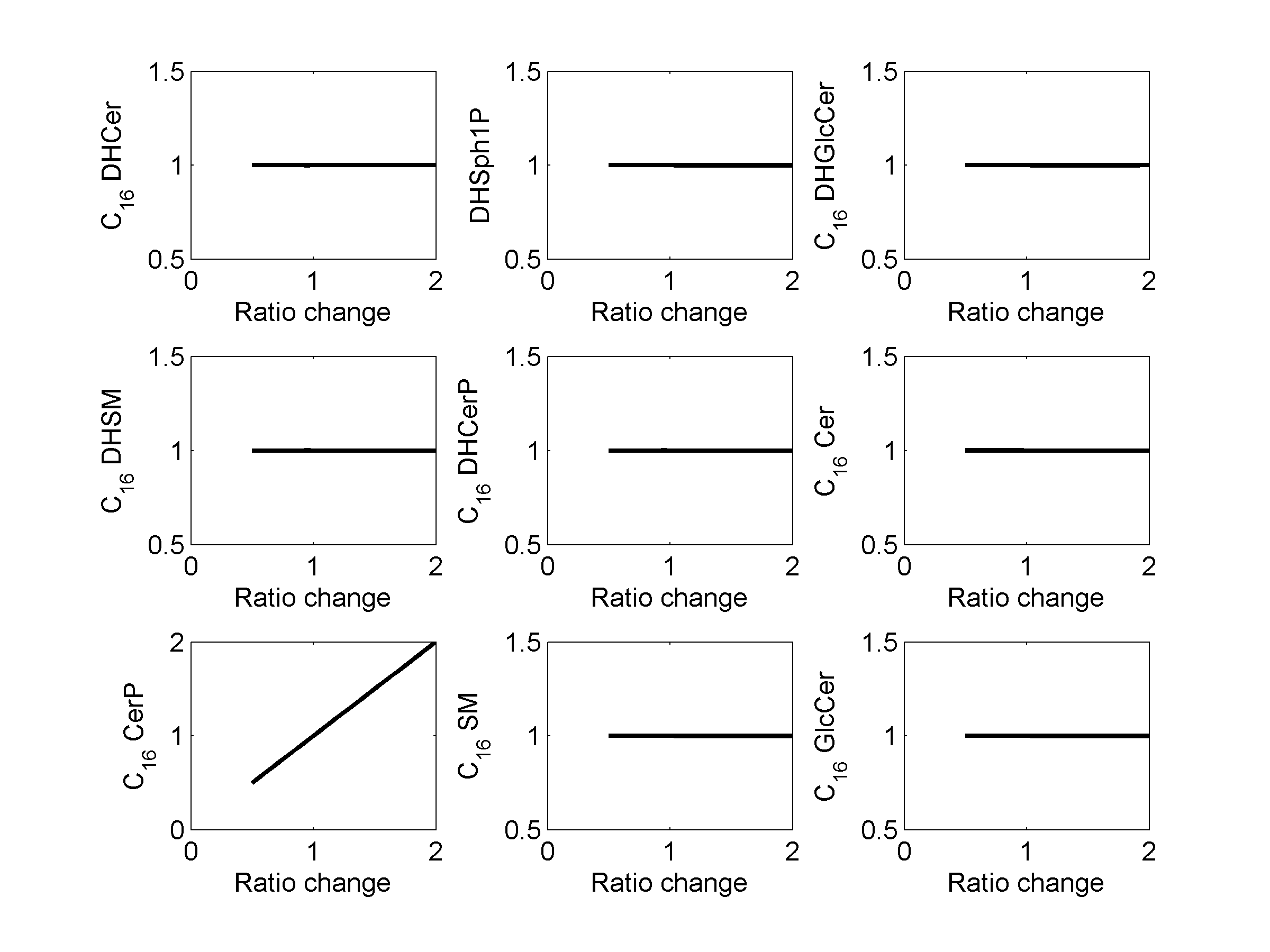
**

**Figure S2.** Simulation results of parametric sensitivity analysis for the parameter k_f17_ (C_16_ Cer + Cerk 🡪 C_16_ CerP). X-axis: ratio of perturbed value of the parameter to the original (optimized) value of the parameter; Y-axis: fold-change in the maximum-value of state variables (metabolites).

**Table S1a.** Microarray fold change data with respect to the control data for genes used in the simulation as input in the network.

| Time Gene | 0.5 hr | 1 hr | 1.5 hr | 2 hr | 4 hr | 6 hr | 8 hr | 12 hr | 18 hr | 24 hr |
| --- | --- | --- | --- | --- | --- | --- | --- | --- | --- | --- |
| Cerk | 0.95 | 0.86 | 0.78 | 0.64 | 0.41 | 0.64 | 0.56 | 0.81 | 1.52 | 1.58 |
| CerS5 | 1.06 | 1.08 | 1.1 | 1.08 | 1 | 1.03 | 1.11 | 1.05 | 0.93 | 0.99 |
| CerS6 | 1.06 | 1.15 | 1.3 | 1.52 | 3.19 | 3.01 | 3.29 | 2.17 | 1.62 | 1.63 |
| Degs1 | 1.1 | 1.1 | 1.23 | 1.33 | 1.82 | 1.89 | 1.74 | 1.85 | 1.9 | 2.02 |
| Degs2 | 0.89 | 0.89 | 0.95 | 0.99 | 1.02 | 1.02 | 1 | 0.97 | 1.02 | 0.94 |
| Smpd1 | 1.08 | 1.05 | 0.98 | 0.9 | 0.81 | 1.37 | 1.17 | 1.77 | 2.44 | 2.6 |
| Sms1 | 1.45 | 1.49 | 1.75 | 1.85 | 1.78 | 1.56 | 1.55 | 1.43 | 1.56 | 1.57 |
| Sms2 | 1.12 | 1.31 | 1.53 | 1.92 | 1.44 | 0.98 | 1.23 | 1.05 | 1.08 | 1.37 |
| Sphk1 | 0.95 | 1.32 | 1.6 | 1.92 | 1.5 | 1.59 | 1.81 | 1.51 | 1.25 | 1.4 |
| Sphk2 | 0.97 | 0.86 | 0.78 | 0.77 | 1.4 | 2.33 | 2.3 | 2.56 | 3.28 | 3.31 |
| Ugcg | 1.1 | 1.41 | 1.43 | 1.63 | 2.32 | 2.62 | 2.53 | 2.2 | 2.21 | 2.2 |

**Table S1b.** Percentage standard error of the mean in the microarray fold change data with respect to the control data for genes used in the simulation as input in the network.

| Time Gene | 0.5 hr | 1 hr | 1.5 hr | 2 hr | 4 hr | 6 hr | 8 hr | 12 hr | 18 hr | 24 hr |
| --- | --- | --- | --- | --- | --- | --- | --- | --- | --- | --- |
| Cerk | 0.06 | 0.07 | 0.07 | 0.11 | 0.15 | 0.25 | 0.16 | 0.19 | 0.13 | 0.11 |
| CerS5 | 0.04 | 0.05 | 0.03 | 0.05 | 0.04 | 0.02 | 0.04 | 0.06 | 0.03 | 0.02 |
| CerS6 | 0.05 | 0.07 | 0.06 | 0.05 | 0.12 | 0.12 | 0.18 | 0.23 | 0.09 | 0.08 |
| Degs1 | 0.04 | 0.05 | 0.06 | 0.06 | 0.04 | 0.02 | 0.04 | 0.04 | 0.04 | 0.04 |
| Degs2 | 0.11 | 0.11 | 0.05 | 0.02 | 0.09 | 0.06 | 0.05 | 0.06 | 0.02 | 0.11 |
| Smpd1 | 0.06 | 0.07 | 0.06 | 0.08 | 0.04 | 0.30 | 0.09 | 0.11 | 0.07 | 0.09 |
| Sms1 | 0.11 | 0.04 | 0.09 | 0.07 | 0.08 | 0.09 | 0.08 | 0.05 | 0.06 | 0.06 |
| Sms2 | 0.13 | 0.15 | 0.11 | 0.11 | 0.04 | 0.08 | 0.08 | 0.08 | 0.07 | 0.10 |
| Sphk1 | 0.10 | 0.25 | 0.24 | 0.30 | 0.32 | 0.22 | 0.17 | 0.11 | 0.08 | 0.06 |
| Sphk2 | 0.05 | 0.03 | 0.03 | 0.03 | 0.03 | 0.13 | 0.01 | 0.02 | 0.05 | 0.06 |
| Ugcg | 0.06 | 0.06 | 0.06 | 0.05 | 0.04 | 0.06 | 0.05 | 0.06 | 0.03 | 0.04 |

**Table S2.** Results of parametric sensitivity analysis*. The sensitivity was calculated as the slope of the sensitivity curves (Fig. 3) at the optimized value of the parameters.

* Unit of parameter sensitivity is fold change in maximum value of metabolites / ratio change in parameter value.

|  | DHCer | DHSph1P | DHGlcCer | DHSM | DHCerP | Cer | CerP | SM | GlcCer |
| --- | --- | --- | --- | --- | --- | --- | --- | --- | --- |
| kf1 | 0.96 | 0.00 | 0.89 | 1.26 | 0.98 | 0.54 | 0.46 | 1.06 | 0.32 |
| kf2 | 0.00 | 0.00 | 0.00 | 0.00 | 0.00 | 0.00 | 0.00 | 0.00 | 0.00 |
| kf3 | 0.00 | 0.00 | 0.00 | 0.00 | 0.00 | 0.00 | 0.00 | 0.00 | 0.00 |
| kf4 | 0.00 | 0.01 | 0.00 | 0.00 | -0.01 | 0.00 | 0.00 | 0.00 | 0.00 |
| kf5 | 0.00 | 0.99 | 0.00 | 0.00 | 0.00 | 0.00 | 0.00 | 0.00 | 0.00 |
| kf6 | 0.00 | -0.93 | 0.00 | 0.00 | 0.00 | 0.00 | 0.00 | 0.00 | 0.00 |
| kf7 | -0.04 | 0.00 | 0.97 | -0.05 | -0.05 | -0.02 | -0.02 | -0.04 | -0.01 |
| kf8 | 0.00 | 0.00 | -0.90 | 0.00 | 0.00 | 0.00 | 0.00 | 0.00 | 0.00 |
| kf9 | -0.08 | 0.00 | -0.08 | 0.24 | -0.05 | -0.06 | -0.06 | -0.10 | -0.06 |
| kb9 | 0.19 | 0.00 | 0.20 | -0.56 | 0.14 | 0.14 | 0.15 | 0.23 | 0.13 |
| kf10 | -0.19 | 0.00 | -0.21 | 0.54 | -0.13 | -0.15 | -0.16 | -0.22 | -0.15 |
| kb10 | 0.07 | 0.00 | 0.08 | -0.18 | 0.05 | 0.05 | 0.06 | 0.08 | 0.05 |
| kf11 | 0.02 | 0.00 | 0.02 | 0.00 | 0.01 | 0.02 | 0.02 | 0.00 | 0.02 |
| kf12 | -0.10 | 0.00 | -0.12 | -0.01 | -0.11 | -0.07 | -0.06 | -0.10 | -0.04 |
| kf13 | 0.00 | 0.00 | 0.00 | 0.00 | 1.00 | 0.00 | 0.00 | 0.00 | 0.00 |
| kf14 | 0.00 | 0.00 | 0.00 | 0.00 | -0.40 | 0.00 | 0.00 | 0.00 | 0.00 |
| kf15 | -0.01 | 0.00 | -0.01 | -0.02 | -0.02 | 0.00 | 0.00 | 0.01 | 0.00 |
| kf16 | -0.64 | 0.00 | -0.62 | -0.64 | -0.74 | 0.25 | 0.26 | 0.41 | 0.24 |
| kf17 | 0.00 | 0.00 | 0.00 | 0.00 | 0.00 | 0.00 | 1.00 | 0.00 | 0.00 |
| kf18 | 0.00 | 0.00 | 0.00 | 0.00 | 0.00 | 0.00 | -0.40 | 0.00 | 0.00 |
| kf19 | 0.00 | 0.00 | 0.00 | 0.00 | 0.00 | -0.14 | -0.15 | 0.04 | -0.15 |
| kb19 | 0.00 | 0.00 | 0.00 | 0.00 | 0.01 | 0.04 | 0.04 | -0.01 | 0.04 |
| kf20 | 0.00 | 0.00 | 0.00 | 0.00 | 0.00 | -0.72 | -0.73 | 0.21 | -0.75 |
| kb20 | 0.00 | 0.00 | 0.00 | 0.00 | 0.01 | 0.81 | 0.81 | -0.23 | 0.82 |
| kf21 | 0.00 | 0.00 | 0.00 | 0.00 | 0.00 | 0.00 | 0.00 | 0.00 | 0.00 |
| kf22 | 0.00 | 0.00 | 0.00 | 0.00 | 0.00 | -0.13 | -0.12 | -0.04 | -0.10 |
| kf23 | 0.00 | 0.00 | 0.00 | 0.00 | 0.00 | 0.00 | 0.00 | 0.00 | 0.00 |
| kf24 | 0.00 | 0.00 | 0.00 | 0.00 | 0.01 | -0.06 | -0.05 | -0.12 | 0.96 |
| kf25 | 0.00 | 0.00 | 0.00 | 0.00 | 0.00 | 0.00 | 0.00 | 0.00 | -0.04 |

**Table S3.** The estimated values of the rate parameters in the model of sphingolipid metabolism where parameters for CerK, Ugcg and SMS1/2 are same for the reactions involving Cer and DHCer.

| No. | Reactions | Parameter Names | Values |
| --- | --- | --- | --- |
| 1 | DHSph + CoA16 + CerS6 🡪 C_16_ DHCer | k_f1_ | 1.30E+01 |
| 2 | DHSph + CoA16 🡪 C_16_ DHCer | k_f2_ | 5.94E-02 |
| 3 | C_16_ DHCer 🡪 | k_f3_ | 1.06E-03 |
| 4 | DHSph + Sphk1 🡪 DHSph1P | k_f4_ | 3.74E-04 |
| 5 | DHSph + Sphk2 🡪 DHSph1P | k_f5_ | 1.57E-02 |
| 6 | DHSph1P 🡪 | k_f6_ | 5.20E-01 |
| 7 | C_16_ DHCer + Ugcg 🡪 C_16_ DHGlcCer | k_f7_ | 2.02E-02 |
| 8 | C_16_ DHGlcCer 🡪 | k_f8_ | 2.90E-01 |
| 9 | C_16_ DHCer + Sms1 + C_16_ GPCho 🡨🡪 C_16_ DHSM + Sms1 + C_16_ DG | k_f9_ k_b9_ | 1.16E-01 1.7201 |
| 10 | C_16_ DHCer + Sms2 + C_16_ GPCho 🡨🡪 C_16_ DHSM + Sms2 + C_16_ DG | k_f10_ k_b10_ | 3.90E-01 8.61E-01 |
| 11 | C_16_ DHSM + Smpd1 🡪 C_16_ DHCer | k_f11_ | 1.08E-01 |
| 12 | C_16_ DHSM 🡪 | k_f12_ | 3.00E-02 |
| 13 | C_16_ DHCer + Cerk 🡪 C_16_ DHCerP | k_f13_ | 1.18E-04 |
| 14 | C_16_ DHCerP 🡪 | k_f14_ | 1.66E-01 |
| 15 | C_16_ DHCer + Degs1 🡪 C_16_ Cer | k_f15_ | 9.25E-03 |
| 16 | C_16_ DHCer 🡪 C_16_ Cer | k_f16_ | 8.00E-01 |
| 17 | C_16_ Cer + Cerk 🡪 C_16_ CerP | k_f17_ | 1.18E-04 |
| 18 | C_16_ CerP 🡪 | k_f18_ | 1.13E-01 |
| 19 | C_16_ Cer + Sms1 + C_16_ GPCho 🡨🡪 C_16_ SM + Sms1 + C_16_ DG | k_f19_ k_b19_ | 1.16E-01 5.59E-02 |
| 20 | C_16_ Cer + Sms2 + C_16_ GPCho 🡨🡪 C_16_ SM + Sms2 + C_16_ DG | k_f20_ k_b20_ | 3.90E-01 1.3469 |
| 21 | C_16_ SM + Smpd1 🡪 C_16_ Cer | k_f21_ | 1.67E-03 |
| 22 | C_16_ SM 🡪 | k_f22_ | 1.06E-02 |
| 23 | C_16_ Cer 🡪 | k_f23_ | 0.00E+00 |
| 24 | C_16_ Cer + Ugcg 🡪 C_16_ GlcCer | k_f24_ | 2.02E-02 |
| 25 | C_16_ GlcCer 🡪 | k_f25_ | 3.17E-03 |
| Microarray data was used for protein concentration. | | | |
| X 🡪 means default degradation of the metabolite X | | | |
| Unit of first order reaction is 1/hr | | | |
| Unit of second order reaction is 1/hr when it involves gene as a modifier as we have used fold change data with respect to control for these variables. | | | |
| Unit of third order reaction is μg DNA/(pmol * hr) when it involves protein as a modifier. | | | |
| Unit of third order reaction is μg DNA/(Ratio Int * hr) when it involves DG and protien as modifiers. | | | |
